# Blarcamesine improves EEG biomarkers of cortical dysfunction in a mouse model of fragile X syndrome

**DOI:** 10.3389/fphar.2026.1786406

**Published:** 2026-09-16

**Authors:** Carrie R. Jonak, Samantha A. Assad, Devin K. Binder, James Tran, Christopher U. Missling, Walter E. Kaufmann

**Affiliations:** 1 Division of Biomedical Sciences, School of Medicine, University of California, Riverside, CA, United States; 2 Neuroscience Graduate Program, University of California, Riverside, CA, United States; 3 Anavex Life Sciences Corp., New York, NY, United States; 4 Department of Human Genetics, Emory University School of Medicine, Atlanta, GA, United States; 5 Department of Neurology, Boston Children’s Hospital, Boston, MA, United States

**Keywords:** biomarker, blarcamesine, electroencephalography, fragile X syndrome, sigma-1 receptor

## Abstract

**Background:**

Fragile X syndrome (FXS) is the most common inherited form of intellectual disability and autism spectrum disorder. Previous studies in the *Fmr1* knockout (KO) mouse model of FXS and affected individuals have identified common electroencephalographic (EEG) abnormalities, which constitute biomarkers of cortical dysfunction for drug treatments.

**Methods:**

We evaluated the effects of three different doses of oral blarcamesine, a Sigma-1 receptor agonist (S1R), on FXS-relevant EEG biomarkers in *Fmr1* KO mice utilizing a multielectrode array (MEA) on resting-state power (from delta to gamma frequencies) and network synchronization (inter-trial phase coherence [ITPC]) using three auditory stimulation paradigms (chirp, auditory steady-state response [ASSR] at 40 Hz and at 80 Hz).

**Results:**

Blarcamesine-treated *Fmr1* KO mice showed dose-dependent modulation of EEG biomarkers across cortical regions. Resting-state EEG power was reduced across multiple frequency bands, including suppression of the FXS-distinctive elevation in the gamma band. Significant improvements in ITPC were observed in all cortical regions for chirp stimulation and 40 Hz ASSR, while modest improvements were seen for 80 Hz ASSR.

**Conclusion:**

Altogether, these data demonstrate that blarcamesine modulates multiple EEG biomarkers of cortical dysfunction in *Fmr1* KO mice. Given that these biomarkers are associated with fundamental neurophysiological mechanisms underlying cognitive and behavioral abnormalities, and are shared between FXS mouse models and affected individuals, these findings are consistent with previously characterized S1R-mediated pharmacological effects of blarcamesine. These data support further investigation of blarcamesine’s clinical potential in FXS and other neurodevelopmental disorders.

## Introduction

Fragile X Syndrome (FXS) is the most common inherited form of intellectual disability and autism spectrum disorder, affecting approximately 1/4,000 males and 1/6,000–1/8,000 females in the United States (Hagerman et al., 2017). FXS is caused from a trinucleotide expansion of a CGG repeat in the 5′ untranslated region of the X chromosome-located *FMR1* gene, which encodes the Fragile X Messenger Ribonucleoprotein 1 (FMRP) (Hagerman et al., 2017; Tassone et al., 2012). Large expansions (>200 repeats), termed full mutation or simply FXS, lead to atypical gene methylation and transcriptional silencing with the consequent reduction to absence of FMRP. Smaller expansions (55–200 repeats) are termed premutation and are not associated with gene silencing but with messenger ribonucleic acid (mRNA) accumulation and other neurologic and endocrine phenotypes (Tassone et al., 2012; Hagerman et al., 2017). As an X-linked disorder, males are more affected than females) (Loesch et al., 2004; Hagerman et al., 2017; Budimirovic et al., 2020). FMRP is an RNA-binding protein that, through its regulation of protein synthesis, plays a key role in synaptic development and function (Hagerman et al., 2017).

Individuals with FXS frequently present with intellectual disability (>90% of males, approximately 50% of females), characteristic physical features, and behavioral abnormalities (Hagerman et al., 2017). Among the latter are anxiety, attention-deficit/hyperactivity disorder (ADHD) features, irritability, aggression, agitation and self-injury (IAAS) behaviors, increased sensory reactivity, and stereotypic and perseverative behavior (Boyle and Kaufmann, 2010; Kidd et al., 2014; Hagerman et al., 2017; Sherman et al., 2017; Kaufmann et al., 2024a; Lachiewicz et al., 2024). A large proportion of affected individuals display autistic features, with 20%–50% meeting diagnostic criteria for autism spectrum disorder (ASD) (approximately 50% of males and 17% of females) (Hagerman et al., 2017; Boyle and Kaufmann, 2010; Kaufmann et al., 2017). Language and other specific cognitive impairments can also be present as well as sleep problems and seizures (Hagerman et al., 2017; Sherman et al., 2017; Berry-Kravis et al., 2021a; Budimirovic et al., 2022). In relationship with the aforementioned sensory symptoms, EEG analyses in FXS have shown increased sensory reactivity and reduced habituation to repeated auditory stimuli (Castrén et al., 2003; Schneider et al., 2013; Ethridge et al., 2016; Ethridge et al., 2017; Ethridge et al., 2019). Moreover, abnormalities in EEG parameters like gamma power are also associated with more severe overall cognitive and behavioral impairment (Ethridge et al., 2019).

As with other neurodevelopmental disorders, FXS is considered a synaptic disorder characterized by abnormalities in homeostatic and Hebbian plasticity, including enhanced long-term depression associated with excessive group I metabotropic glutamate receptor (mGluR) signaling (Kaufmann et al., 2024b). Several therapeutic strategies have been investigated in FXS, reflecting the complex and multifactorial nature of the disorder. Modulation of excessive glutamatergic signaling through mGluR5 antagonism has been extensively studied, with preclinical studies demonstrating correction of multiple FXS-relevant phenotypes in *Fmr1* KO mice (Michalon et al., 2012; Berry-Kravis et al., 2018). However, translation into clinical benefit has proven challenging, as exemplified by the lack of efficacy observed in clinical trials of mavoglurant (Berry-Kravis et al., 2016; Berry-Kravis et al., 2018). Approaches targeting impaired inhibitory neurotransmission have also shown promise. Arbaclofen, a GABA-B receptor agonist, improved behavioral and electrophysiological phenotypes in *Fmr1* KO mice, although subsequent clinical studies yielded mixed results (Berry-Kravis et al., 2017; Berry-Kravis et al., 2018). Additional therapeutic approaches have targeted downstream signaling pathways and excitatory-inhibitory balance, including lovastatin, which corrected excessive protein synthesis and epileptogenesis-related phenotypes in preclinical models (Osterweil et al., 2013), and modulation of the endocannabinoid system, which has been implicated in synaptic dysfunction associated with FXS (Busquets-Garcia et al., 2013). More recently, a trial of a phosphodiesterase-4D inhibitor demonstrated improvements in cognition in adults with FXS (Berry-Kravis et al., 2021b), raising the possibility of disease-specific therapeutic interventions.

Despite these encouraging findings, the FXS field would benefit from additional pharmacological approaches targeting broader mechanisms of cellular and synaptic homeostasis. Novel therapeutic strategies in neurodevelopmental disorders increasingly focus on cellular homeostasis and multiple signaling pathways as potential therapeutic targets (Gantois et al., 2006; Cogram et al., 2022; Kaufmann et al., 2024b). Among these drugs is trofinetide, a peptide derived from IGF-1 (Parent et al., 2023), which showed some benefits in an early clinical trial (Berry-Kravis et al., 2020). Another type of compound that can broadly affect cellular homeostasis and signaling is sigma-1 receptor (S1R) modulators, which have shown promise in Alzheimer’s disease clinical trials (Hampel et al., 2020; Macfarlane et al., 2025).

The S1R is an intracellular chaperone protein located at the endoplasmic reticulum-mitochondria interface, where it regulates calcium flux between these two organelles (Su et al., 2016; Schmidt and Kruse, 2019). Activation of the S1R leads to, among other effects, improvements in mitochondrial function, promotion of autophagy and decreases in levels of reactive oxygen species and neuroinflammation (Christ et al., 2019; Malar et al., 2023). At the physiological level, mitochondrial calcium influx regulates hippocampal firing rate and homeostatic synaptic plasticity (Styr et al., 2019). Administration of a S1R agonist has corrected maladaptive homeostatic synaptic scaling in a mouse model of Huntington disease (Smith-Dijak et al., 2019). The S1R is widely distributed in the brain, with relatively higher density in the frontal cortex, hippocampus, cerebellum, and some diencephalic and brainstem nuclei (Reyes et al., 2021). Two previous preclinical studies of the S1R agonist, blarcamesine, support its use in FXS and other neurodevelopmental disorders (Kaufmann et al., 2019; Reyes et al., 2021; Cogram et al., 2022).

A combination of positron emission tomography with the highly selective S1R ligand [^18^F]FTC-146 and *ex vivo* autoradiographic studies demonstrated the drug’s dose-dependent receptor occupancy (Reyes et al., 2021). It also showed that the broad but variable regional distribution of the S1R in wild-type (WT) mice is preserved in a widely used FXS mouse model (i.e., *Fmr1* KO mice). Furthermore, administration of blarcamesine to 2-month-old *Fmr1* KO mice for 2 weeks led to, in comparison with WT animals, normalization of performance on the open field and contextual fear conditioning tests and improvement in marble-burying behavior. These are key neurobehavioral phenotypes which model, respectively, hyperactivity, associative learning, and anxiety and perseverative behavior in FXS (Reyes et al., 2021). The same blarcamesine treatment also restored levels of hippocampal BDNF (Reyes et al., 2021), a major regulator of synaptic signaling, and decreased abnormal elevations of a marker of phosphatidylinositol 3-kinase/Protein Kinase B/mechanistic target of rapamycin (PI3K/Akt/mTOR) activation in peripheral lymphocytes (Cogram et al., 2022). Altogether, these data showed that doses representing measurable S1R occupancy are effective for improving key cell signaling and behavioral phenotypes in *Fmr1* KO mice.

The potential of EEG parameters as biomarkers of treatment response in both individuals with FXS and related animal models has been established in several preclinical and clinical studies testing different drugs (Lovelace et al., 2020; Berry-Kravis et al., 2021b; Jonak et al., 2021; Jonak et al., 2022; Pirbhoy et al., 2021). Recent investigations have relied on multielectrode array (MEA) analyses in *Fmr1* KO mice developed by us (Jonak et al., 2018; Jonak et al., 2020; Jonak et al., 2024). The system involves chronic *in vivo* implantation of a planar MEA on the surface of the mouse skull and 30-channel simultaneous EEG, with acquisition of resting and stimulus-evoked EEG in awake, freely moving animals. Electrophysiological methods compatible with analyses in humans and mice have demonstrated marked abnormalities in resting EEG power, particularly in the gamma frequency band (Bakker et al., 1994; Lovelace et al., 2020; Jonak et al., 2021; Jonak et al., 2022). In addition, auditory stimulation paradigms, namely, chirp and auditory steady-state response (ASSR) at low and high gamma frequencies, have shown anomalous evoked responses to single-trial and train-related EEG power and reduced inter-trial phase coherence (ITPC) in *Fmr1* KO mice (Jonak et al., 2024).

Taking advantage of these novel EEG biomarker tools for drug development research, directly translatable to individuals with FXS, the present study extends our previous testing of blarcamesine in *Fmr1* KO mice (Reyes et al., 2021; Cogram et al., 2022) by evaluating the effects of different doses of blarcamesine on validated EEG biomarkers. Specifically, we analyzed resting state power (from delta to gamma frequencies) and network synchronization (ITPC) on the chirp, 40 Hz ASSR and 80 Hz ASSR auditory stimulation paradigms in *Fmr1* KO mice before and after the administration of vehicle or blarcamesine at three different doses. Resting-state and post-auditory stimulation recordings, including comparisons between WT and KO mice and among blarcamesine treatment groups, demonstrated dose-dependent modulation of EEG biomarkers, supporting further investigation of blarcamesine as a potential therapeutic approach for individuals with FXS.

## Methods

### Animals and experimental design

All procedures were approved by the Institutional Animal Care and Use Committee at the University of California, Riverside and in accordance with the NIH Animal Care and Use Guidelines. The mice used in this study were male WT and *Fmr1* KO mice on a C57BL/6J background maintained in-house. The colony was established from breeding pairs of *Fmr1* KO (B6.129P2-Fmr1tm1Cgr/J, stock #003025) and C57BL/6J WT (stock #000664) from Jackson Laboratory (Bar Harbor, ME). Hemizygous male and homozygous female *Fmr1* KO mice were bred to generate mutant mice. C57BL/6 WT male and WT female mice were bred to generate WT mice. The WT and KO mice were maintained in-house as individual strains, with mice generated grouped-housed in numbers of 2–5 animals per cage. Mice were maintained in an AAALAC-accredited facility under a 12-h light/dark cycle and were provided irradiated rodent diet (PicoLab, 5053) and water *ad libitum*. All genotypes were confirmed by Transnetyx (Cordova, TN) using real-time PCR analysis.

Blarcamesine solutions were suspended using the same saline administered as vehicle. Drug treatment was conducted for 14 days, via daily oral gavage administration, after pre-drug baseline recordings (Figure 1). Group sizes were prospectively determined based on previous experience and published studies using this model (Jonak et al., 2018; Jonak et al., 2020; Jonak et al., 2024), which consistently demonstrated robust genotype-dependent differences between WT and *Fmr1* KO mice with sample sizes of approximately n = 10 per group. Accordingly, the vehicle WT and vehicle *Fmr1* KO groups were assigned n = 10 animals. The 1 mg/kg dose was included as an exploratory low-dose arm to assess whether blarcamesine produced measurable effects at lower exposure levels. Because this group was intended to evaluate the presence of a treatment signal rather than serve as a primary efficacy comparison, a sample size consistent with the control groups (n = 10) was considered appropriate. In contrast, larger sample sizes (n = 15) were prospectively allocated to the 10 and 30 mg/kg treatment groups to increase sensitivity for detecting potential treatment-related effects, given the greater uncertainty regarding the magnitude and variability of responses at these doses. Group sizes were pre-specified prior to study initiation and were not modified based on interim analyses or observed treatment responses.

**FIGURE 1:**
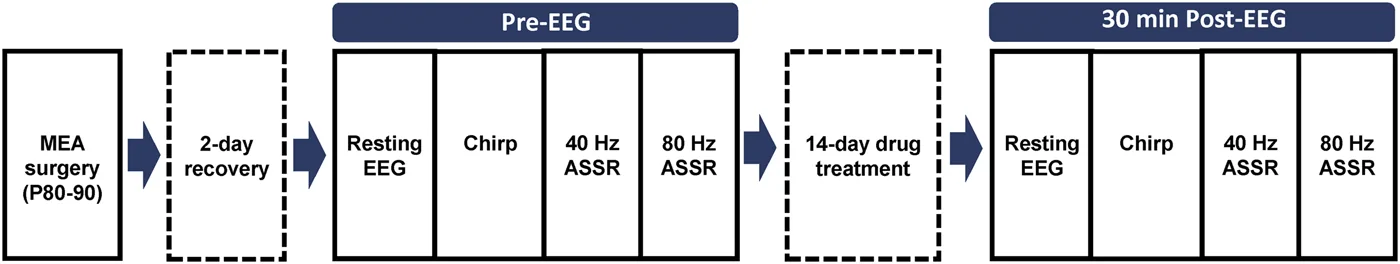
Diagram representing the study design. The protocol included 4 major components: MEA placement and recovery period, Pre-drug treatment EEG recordings, 14‐day drug treatment, and Post‐drug treatment EEG recordings.

### EEG recordings and data analysis

MEA surgical and recording procedures have been previously published (Jonak et al., 2018; Jonak et al., 2020; Jonak et al., 2024). Pre-drug baseline recordings were conducted 2 days after recovery from MEA implantation surgery (unpublished data showed that this post-surgical interval is sufficient for obtaining reliable resting state recordings in WT and *Fmr1* KO mice). Post-drug treatment EEG recordings were done on the same mice 30 min after receiving treatment on day fourteen. Each recording session included resting-state EEG, auditory chirp, and 40 Hz and 80 Hz ASSR stimuli (see details below). Recordings covered left and right frontal, medial, and temporal cortices, as reported in Jonak et al. (2024). EEG data were collected using the SmartBox (Neuronexus) acquisition system from awake and freely moving mice. Acquisition hardware was set to lower (0.5 Hz) and upper (500 Hz) filters and data were sampled at a rate of 1250 Hz. EEG data were analyzed using BrainVision Analyzer 2.1 (Brain Products) together with custom MATLAB scripts and GraphPad Prism. Data were extracted from SmartBox files and converted to a format compatible with Analyzer 2.1. Recordings were downsampled to 625 Hz, and a 60-Hz notch filter was applied. EEG artifacts were removed using a semi-automated procedure, with less than 20% of the data excluded because of artifacts for any individual mouse.

To delineate the electrophysiological phenotype in the current cohort, post-treatment EEG recordings from vehicle-treated *Fmr1* KO mice were compared with post-treatment vehicle-treated WT controls for resting-state EEG power and auditory-evoked responses (chirp, 40 Hz ASSR, and 80 Hz ASSR). To evaluate the effects of blarcamesine, post-treatment EEG recordings from each blarcamesine-treated *Fmr1* KO group (1, 10, and 30 mg/kg) were analyzed separately by comparison with post-treatment vehicle-treated WT controls. This analytical approach allowed genotype-related electrophysiological abnormalities to be established independently of treatment effects while evaluating dose-dependent modulation of EEG biomarkers following blarcamesine administration.

### Resting-state

After habituation, mice underwent a 5-min resting-state EEG recording in the absence of auditory stimulation. Resting-state EEG recordings were segmented into 1-s epochs, and fast Fourier transforms (FFT) were performed using a 10% Hanning window with 0.5-Hz frequency resolution. Average power (μV^2^/Hz) was calculated from 1 to 100 Hz and grouped into the following frequency bands: delta (1–4 Hz), theta (4–8 Hz), alpha (8–13 Hz), beta (13–30 Hz), low gamma (30–55 Hz), and high gamma (65–100 Hz). Data were expressed as ratio values to facilitate comparison across frequency bands using a common scale. Statistical analyses were performed independently for each cortical region using a two-way ANOVA with genotype/treatment group and frequency band as factors. Post hoc multiple comparisons were adjusted using the Šídák-Bonferroni method (α = 0.05) to control the family-wise error rate across six comparisons.

### Auditory stimulation

Following 5 min of resting-state recording, we evaluated the ability of neural generators to produce synchronized oscillations to time varying stimuli (Ethridge et al., 2017) using two types of auditory stimuli. Chirp and ASSR were employed to perform an ITPC analysis (phase locking factor) (Tallon-Baudry et al., 1996; Jonak et al., 2024).

Acoustic stimuli were generated using RPVDSEX software and RZ6 hardware (Tucker Davis Technologies, FL) and presented through a free-field speaker (MF1 Multi-Field Magnetic Speaker; Tucker-Davis Technologies, FL) located 12 in directly above the arena. Sound pressure level (SPL) was modified using programmable attenuators in the RZ6 system. The speaker output was ∼70 dB SPL at the floor of the recording chamber with fluctuation of ±3 dB for frequencies between 5 and 35 kHz as measured with a ¼ inch Bruel & Kjaer microphone. Sound delivery was synchronized with EEG recordings using a TTL pulse to mark the onset of each sound in a train.

### Chirp

The chirp stimulus used was broadband noise whose amplitude was modulated by a sinusoid with linearly increasing frequencies from 1 to 100 Hz (Artieda et al., 2004; Pérez-Alcázar et al., 2008). Each stimulus was 2 s in duration, and the depth of modulation was 100%. Chirp trains were presented via a speaker positioned at the floor of the recording chamber at ∼70 dB SPL 300 times with the interval between each train randomly generated to be between 1 and 1.5 s. We confirmed in each case that this dB level did not induce audiogenic seizures. The chirp facilitates a rapid measurement of transient oscillatory entrainment (delta to gamma frequency range) to auditory stimuli of a wide range of frequencies and can be used to compare oscillatory responses in different groups in clinical and preclinical settings (Purcell et al., 2004). Chirp trains were processed with Morlet wavelets linearly spaced from 1 to 100 Hz using voltage (μV) and wavelet coefficients were exported as complex values for use with ITPC analysis. To measure phase synchronization at each frequency across trials, ITPC was calculated with the following equation:

$$\text{ITPC }\left(f,t\right)=\frac{1}{n}\sum_{k=l}^{n}\frac{F_{k}\left(f,t\right)}{\left|F_{k}\left(f,t\right)\right|}$$

where *f* is the frequency, *t* is the time point, and *k* is trial number. Thus, *F*_*k*_(*f*, *t*) refers to the complex wavelet coefficient at a given frequency and time for the *k*th trial. There were no less than 275 chirp trials (out of 300) for any given mouse after segments containing artifacts were rejected.

### Auditory steady-state response (ASSR)

We used a click train to assess the ASSR, which has been used as a diagnostic biomarker for disorders such as schizophrenia (Brenner et al., 2009; O’Donnell et al., 2013). The ASSR drives steady brain oscillations at specific frequencies of interest. In this study, 40 Hz and 80 Hz gamma frequencies were used to obtain ASSR, with the 40 Hz and 80 Hz generators likely located in cortex and brainstem, respectively (Pastor et al., 2002; Picton et al., 2003a). The ASSR stimulus trains consisted of 0.5 msec clicks repeated at a rate of either 40 or 80 Hz over a 3 s period. Each train was presented 50 times with an inter-train interval of 2 s.

### Chirp and ASSR ITPC analyses

Statistical group comparisons of ITPC in chirp and ASSR (40 Hz and 80 Hz) traces were quantified using a Monte Carlo permutation approach. Analysis was conducted by binning time into 256 parts and frequency into 100 parts, resulting in a 100 × 256 matrix. Non-parametric analysis was used to determine contiguous regions in the matrix that were significantly different from a distribution of 2000 randomized Monte Carlo permutations based on previously published methods (Maris and Oostenveld, 2007). Cluster sizes of the real genotype (both positive and negative direction, resulting in a two-tailed alpha of *p* = 0.025) that were larger than 97.25% of the random group assignments, were considered significantly different between genotypes. This method avoids statistical assumptions about the data and corrects for multiple comparisons.

## Results

### Resting-state and auditory stimulation responses in *Fmr1* KO mice

Consistent with previous studies (Lovelace et al., 2018; Jonak et al., 2024), vehicle-treated *Fmr1* KO mice exhibited the characteristic electrophysiological abnormalities associated with the *Fmr1* KO phenotype. Relative to vehicle-treated WT mice, vehicle-treated *Fmr1* KO mice displayed significantly elevated resting-state low- and high-gamma power (Supplementary Figure S1), together with reduced ITPC during chirp, 40 Hz ASSR, and 80 Hz ASSR stimulation (Supplementary Figures S2–S4). These findings confirmed the expected electrophysiological phenotype in the present cohort and established the reference against which the effects of blarcamesine were evaluated.

### Effects of blarcamesine on resting-state power in *Fmr1* KO mice

Blarcamesine produced dose-dependent modulation of resting-state EEG power, with the significant effects observed within the gamma frequency range. Treatment with 30 mg/kg reduced the elevated low- and high-gamma power characteristic of vehicle-treated *Fmr1* KO mice across all cortical regions, resulting in no significant differences relative to WT mice while producing minimal changes in lower-frequency bands (delta, theta, alpha, and beta) (Figure 2). Treatment with 10 mg/kg produced a similar pattern of gamma power modulation, resulting in no significant differences in all cortical regions relative to WT mice while producing minimal changes in lower-frequency bands (delta, theta, alpha, and beta) (Figure 3). Treatment with 1 mg/kg also improved resting-state EEG power across all cortical regions, with the exception of high-gamma power in the left frontal cortex, which remained significantly different from WT mice (Supplementary Figure S5). Collectively, these findings demonstrate dose-dependent modulation of resting-state gamma power abnormalities by blarcamesine while largely preserving lower-frequency oscillatory activity.

**FIGURE 2:**
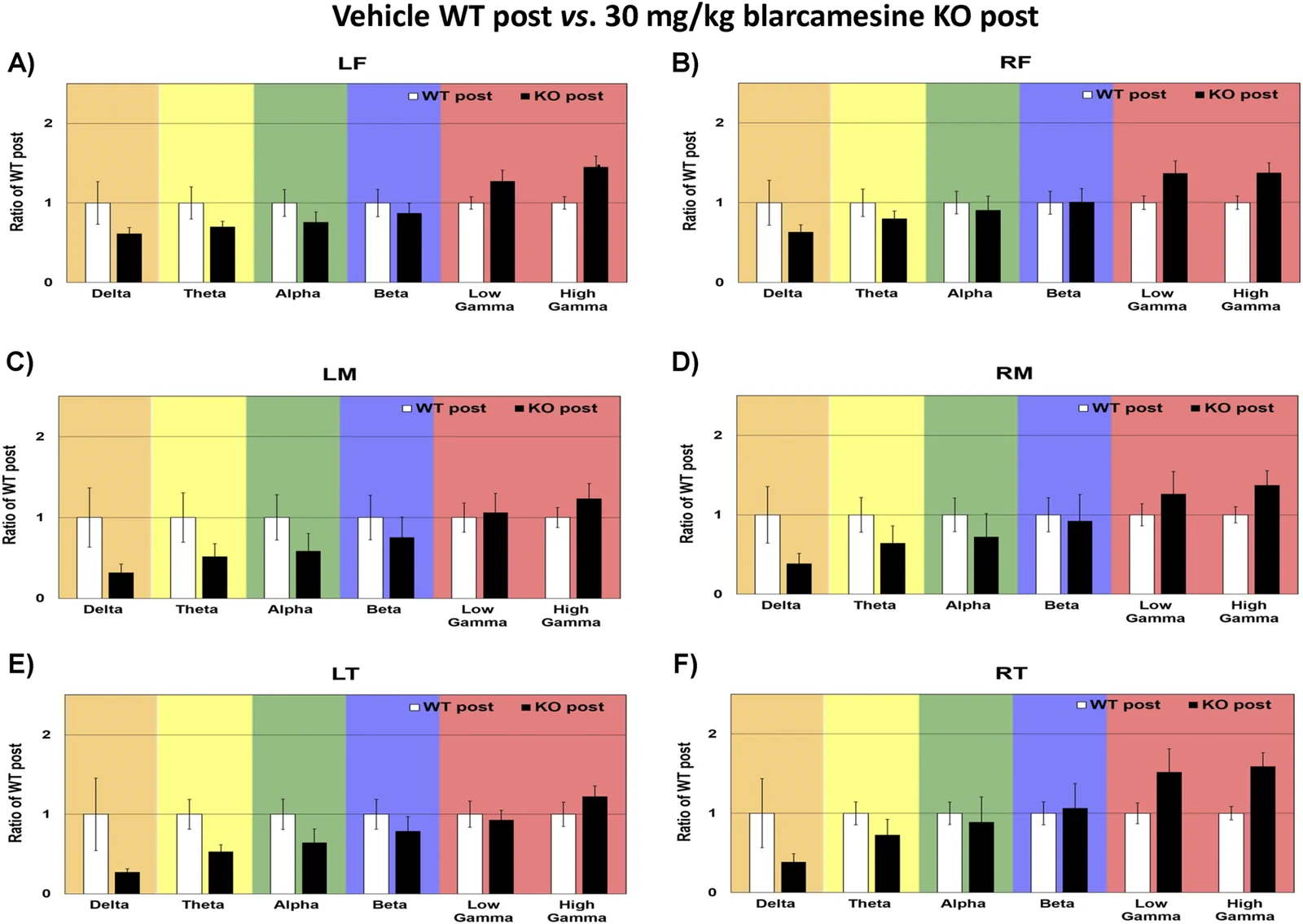
Resting-state power spectral density in vehicle-treated WT vs. 30 mg/kg blarcamesine-treated *Fmr1* KO mice. **(A–F)** Ratios of WT vehicle post-treatment (black bars) to 30 mg/kg blarcamesine-treated *Fmr1* KO mice (white bars) represent EEG resting state power across frequency bands for distinct brain regions. Values are expressed as group means, and error bars represent the standard error of the mean (SEM). Vehicle-treated WT, n = 10; 30 mg/kg blarcamesine-treated *Fmr1* KO, n = 15.

**FIGURE 3:**
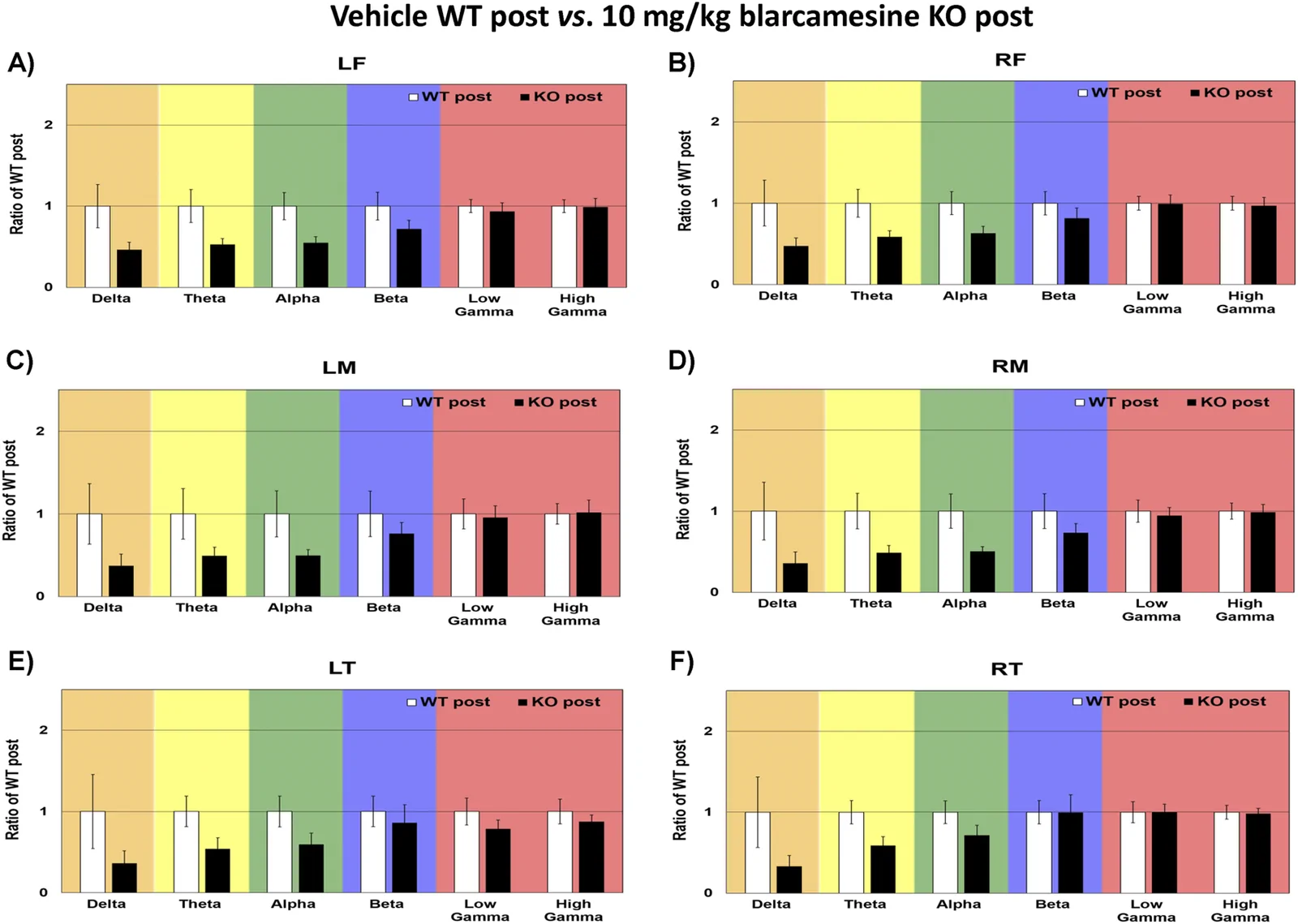
Resting-state power spectral density in vehicle-treated WT vs. 10 mg/kg blarcamesine-treated *Fmr1* KO mice. **(A–F)** Ratios of WT vehicle post-treatment (black bars) to 10 mg/kg blarcamesine-treated *Fmr1* KO mice (white bars) represent EEG resting state power across frequency bands for distinct brain regions. Values are expressed as group means, and error bars represent the standard error of the mean (SEM). Vehicle-treated WT, n = 10; 10 mg/kg blarcamesine-treated *Fmr1* KO, n = 15.

### Effects of blarcamesine on chirp synchronization in *Fmr1* KO mice

Treatment with 30 mg/kg markedly increased chirp ITPC across all cortical regions, resulting in no significant differences relative to WT mice (Figure 4). Treatment with 10 mg/kg also substantially improved ITPC, although limited residual differences relative to WT mice remained in all cortical regions (Figure 5). Treatment with 1 mg/kg produced improvements, resulting in no significant differences relative to WT mice in all cortical regions with the exception of the left and right frontal cortex, which remained significantly different from WT mice (Supplementary Figure S6). Collectively, these findings demonstrate dose-dependent modulation of auditory chirp synchronization by blarcamesine.

**FIGURE 4:**
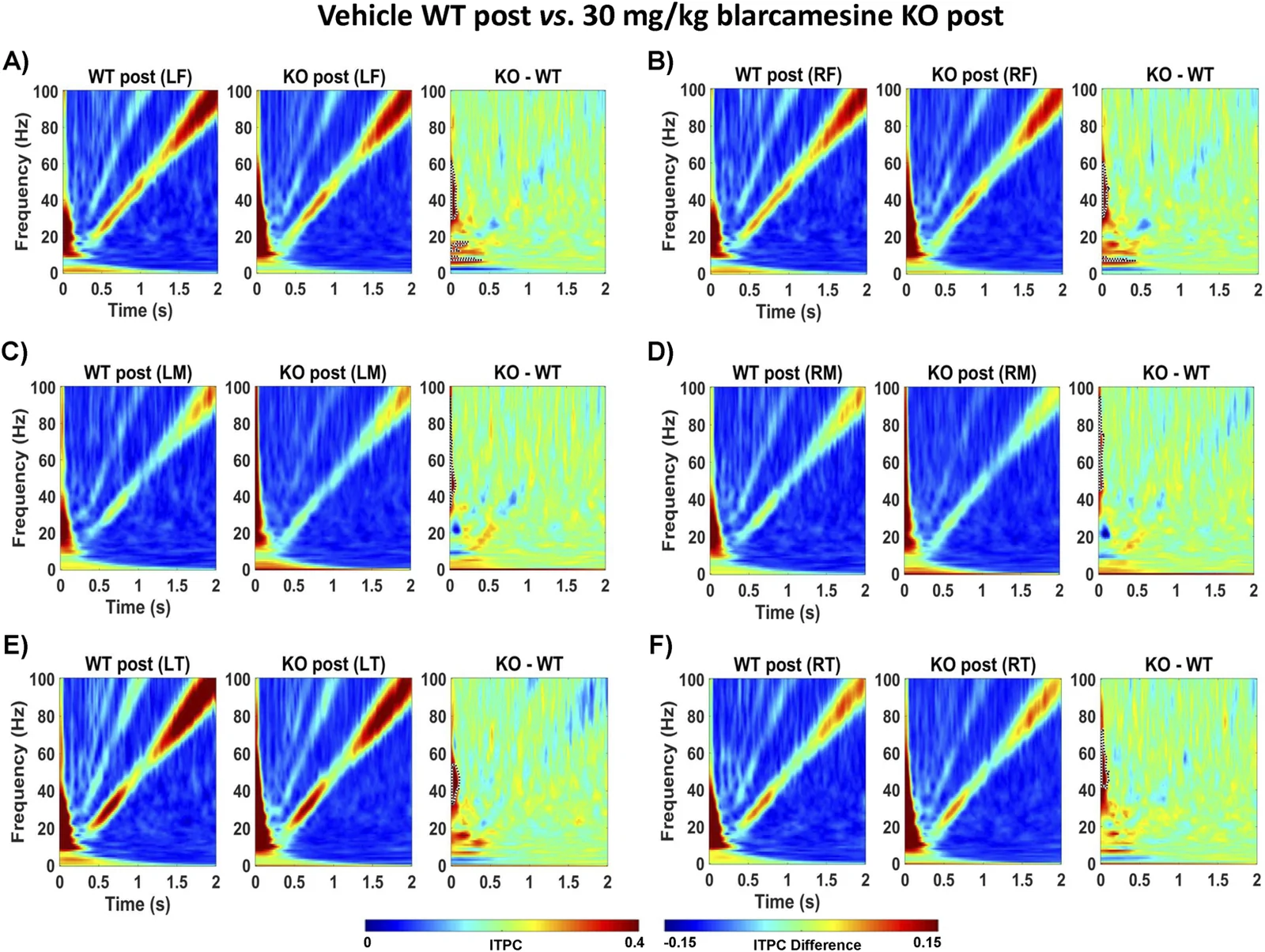
Inter-trial phase coherence (ITPC) to auditory chirp stimulation in vehicle-treated WT vs. 30 mg/kg blarcamesine-treated *Fmr1* KO. **(A–F)** For each brain region, the left panel shows vehicle-treated WT mice, the middle panel shows 30 mg/kg blarcamesine-treated *Fmr1* KO mice, and the right panel shows the difference (*Fmr1* KO – WT). Scales indicate ITPC and ITPC difference. Significant differences are outlined in black. Red indicates greater ITPC in 30 mg/kg blarcamesine-treated *Fmr1* KO mice relative to vehicle-treated WT mice, whereas blue indicates lower ITPC in 30 mg/kg blarcamesine-treated *Fmr1* KO mice relative to vehicle-treated WT mice. LF, left frontal; RF, right frontal; LM, left medial; RM, right medial; LT, left temporal; RT, right temporal. Vehicle-treated WT, n = 10; 30 mg/kg blarcamesine-treated Fmr1 KO, n = 15.

**FIGURE 5:**
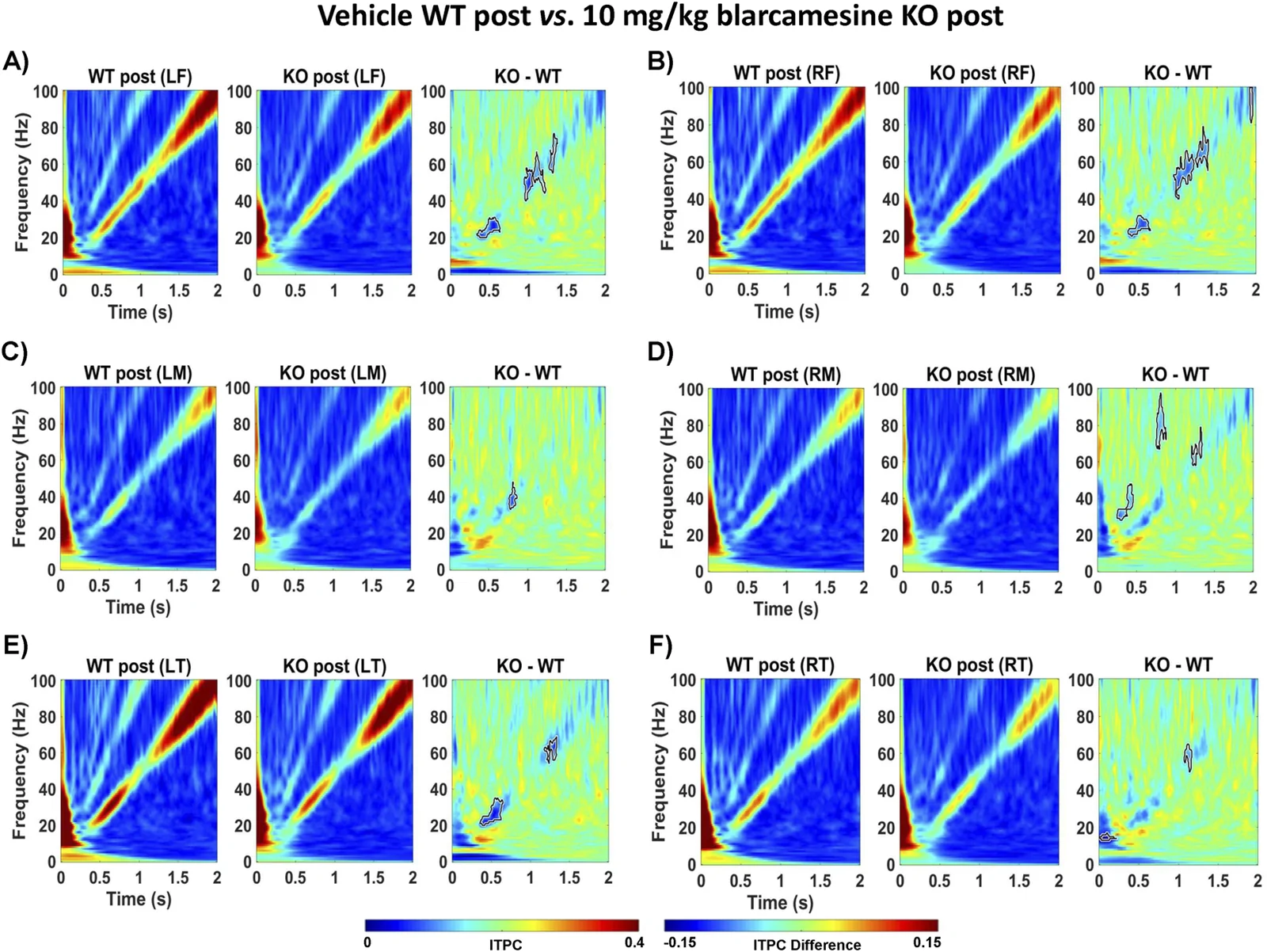
Inter-trial phase coherence (ITPC) to auditory chirp stimulation in vehicle-treated WT vs. 10 mg/kg blarcamesine-treated *Fmr1* KO. **(A–F)** For each brain region, the left panel shows vehicle-treated WT mice, the middle panel shows 10 mg/kg blarcamesine-treated *Fmr1* KO mice, and the right panel shows the difference (*Fmr1* KO – WT). Scales indicate ITPC and ITPC difference. Significant differences are outlined in black. Red indicates greater ITPC in 10 mg/kg blarcamesine-treated *Fmr1* KO mice relative to vehicle-treated WT mice, whereas blue indicates lower ITPC in 10 mg/kg blarcamesine-treated Fmr1 KO mice relative to vehicle-treated WT mice. LF, left frontal; RF, right frontal; LM, left medial; RM, right medial; LT, left temporal; RT, right temporal. Vehicle-treated WT, n = 10; 10 mg/kg blarcamesine-treated *Fmr1* KO, n = 15.

### Effects of blarcamesine on 40 Hz ASSR synchronization in *Fmr1* KO mice

Treatment with 30, 10 and 1 mg/kg markedly increased 40 Hz ASSR ITPC across all cortical regions, resulting in no significant differences relative to WT mice across all cortical regions (Figures 6, 7; Supplementary Figure S7). In addition to the primary response at 40 Hz, significant changes were also observed near 80 Hz, corresponding to the harmonic frequency of the stimulus. Because the physiological significance of this harmonic response remains uncertain, these findings are presented without further interpretation. Collectively, these findings demonstrate modulation of 40 Hz auditory steady-state synchronization by blarcamesine at all tested doses.

**FIGURE 6:**
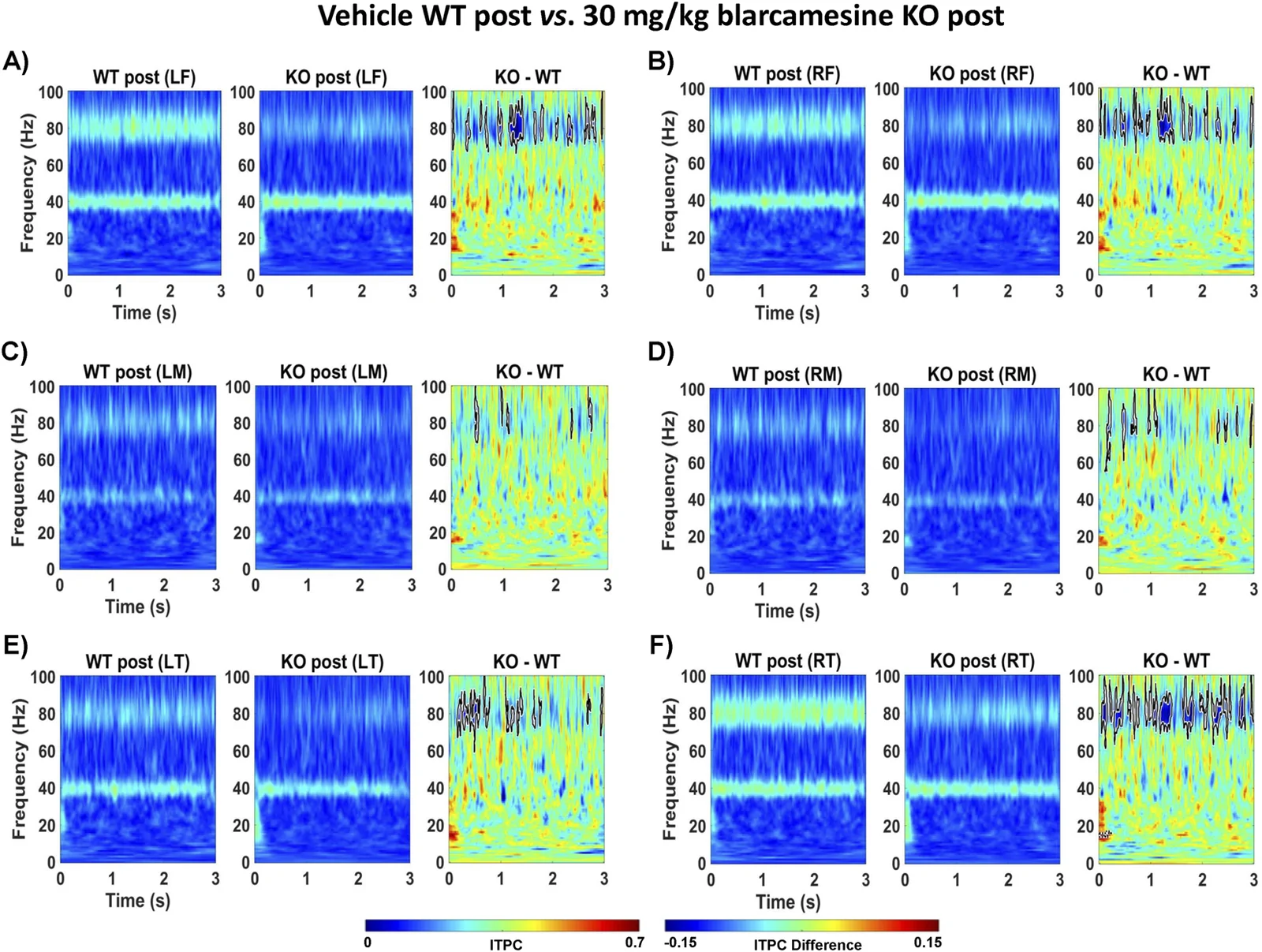
Inter-trial phase coherence (ITPC) to 40 Hz auditory steady-state response (ASSR) in vehicle-treated WT vs. 30 mg/kg blarcamesine-treated *Fmr1* KO. **(A–F)** For each brain region, the left panel shows vehicle-treated WT mice, the middle panel shows 30 mg/kg blarcamesine-treated *Fmr1* KO mice, and the right panel shows the difference (*Fmr1* KO – WT). Scales indicate ITPC and ITPC difference. Significant differences are outlined in black. Red indicates greater ITPC in 30 mg/kg blarcamesine-treated *Fmr1* KO mice relative to vehicle-treated WT mice, whereas blue indicates lower ITPC in 30 mg/kg blarcamesine-treated *Fmr1* KO mice relative to vehicle-treated WT mice. LF, left frontal; RF, right frontal; LM, left medial; RM, right medial; LT, left temporal; RT, right temporal. Vehicle-treated WT, n = 10; 30 mg/kg blarcamesine-treated *Fmr1* KO, n = 15.

**FIGURE 7:**
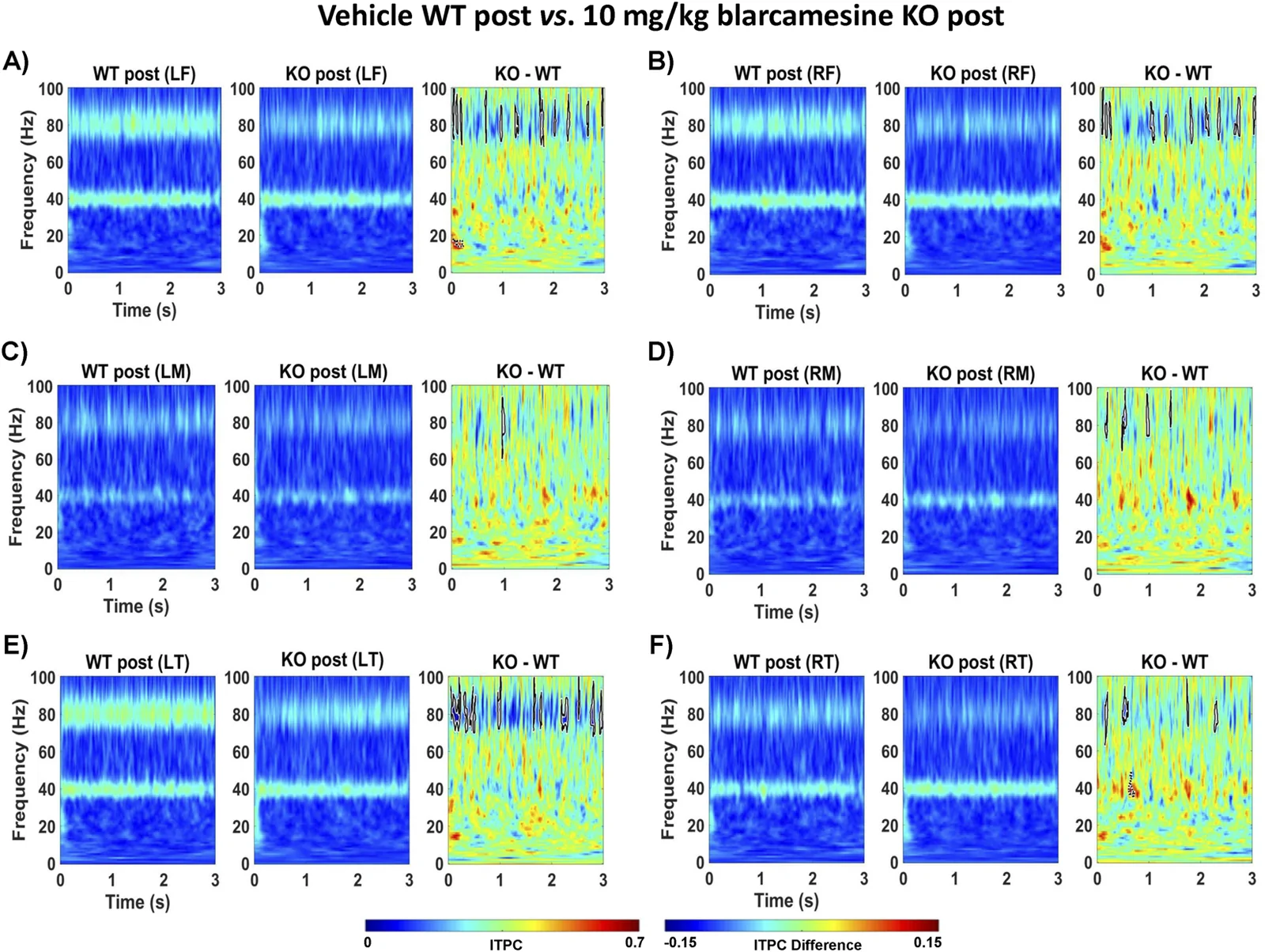
Inter-trial phase coherence (ITPC) to 40 Hz auditory steady-state response (ASSR) in vehicle-treated WT vs. 10 mg/kg blarcamesine-treated *Fmr1* KO. **(A–F)** For each brain region, the left panel shows vehicle-treated WT mice, the middle panel shows 10 mg/kg blarcamesine-treated *Fmr1* KO mice, and the right panel shows the difference (*Fmr1* KO – WT). Scales indicate ITPC and ITPC difference. Significant differences are outlined in black. Red indicates greater ITPC in 10 mg/kg blarcamesine-treated *Fmr1* KO mice relative to vehicle-treated WT mice, whereas blue indicates lower ITPC in 10 mg/kg blarcamesine-treated *Fmr1* KO mice relative to vehicle-treated WT mice. LF, left frontal; RF, right frontal; LM, left medial; RM, right medial; LT, left temporal; RT, right temporal. Vehicle-treated WT, n = 10; 10 mg/kg blarcamesine-treated *Fmr1* KO, n = 15.

### Effects of blarcamesine on 80 Hz ASSR synchronization in *Fmr1* KO mice

Treatment with 30 mg/kg reduced the extent of significant ITPC differences between *Fmr1* KO and WT mice in all cortical regions except the left temporal cortex; however, significant differences remained in every region (Figure 8). Treatment with 10 mg/kg improved ITPC within the left and right medial cortical regions, resulting in no significant differences relative to WT mice in those regions, while significant differences persisted in the frontal and temporal cortices (Figure 9). Treatment with 1 mg/kg produced a similar pattern, with significant differences remaining across the frontal and temporal cortical regions (Supplementary Figure S8). Collectively, these findings indicate that blarcamesine produced only modest modulation of 80 Hz ASSR synchronization.

**FIGURE 8:**
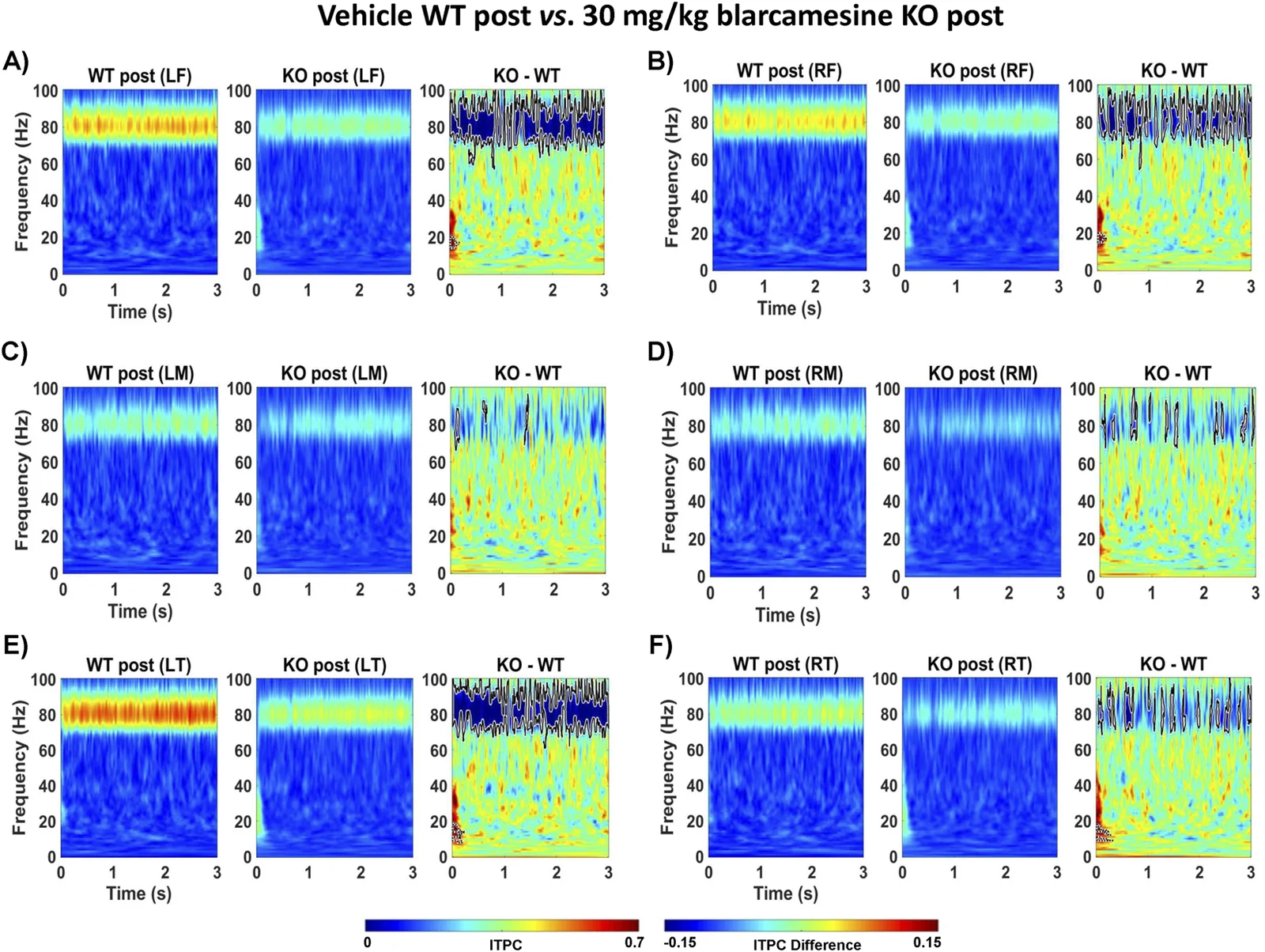
Inter-trial phase coherence (ITPC) to 80 Hz auditory steady-state response (ASSR) in vehicle-treated WT vs. 30 mg/kg blarcamesine-treated *Fmr1* KO. **(A–F)** For each brain region, the left panel shows vehicle-treated WT mice, the middle panel shows 30 mg/kg blarcamesine-treated *Fmr1* KO mice, and the right panel shows the difference (*Fmr1* KO – WT). Scales indicate ITPC and ITPC difference. Significant differences are outlined in black. Red indicates greater ITPC in 30 mg/kg blarcamesine-treated *Fmr1* KO mice relative to vehicle-treated WT mice, whereas blue indicates lower ITPC in 30 mg/kg blarcamesine-treated *Fmr1* KO mice relative to vehicle-treated WT mice. LF, left frontal; RF, right frontal; LM, left medial; RM, right medial; LT, left temporal; RT, right temporal. Vehicle-treated WT, n = 10; 30 mg/kg blarcamesine-treated *Fmr1* KO, n = 15.

**FIGURE 9:**
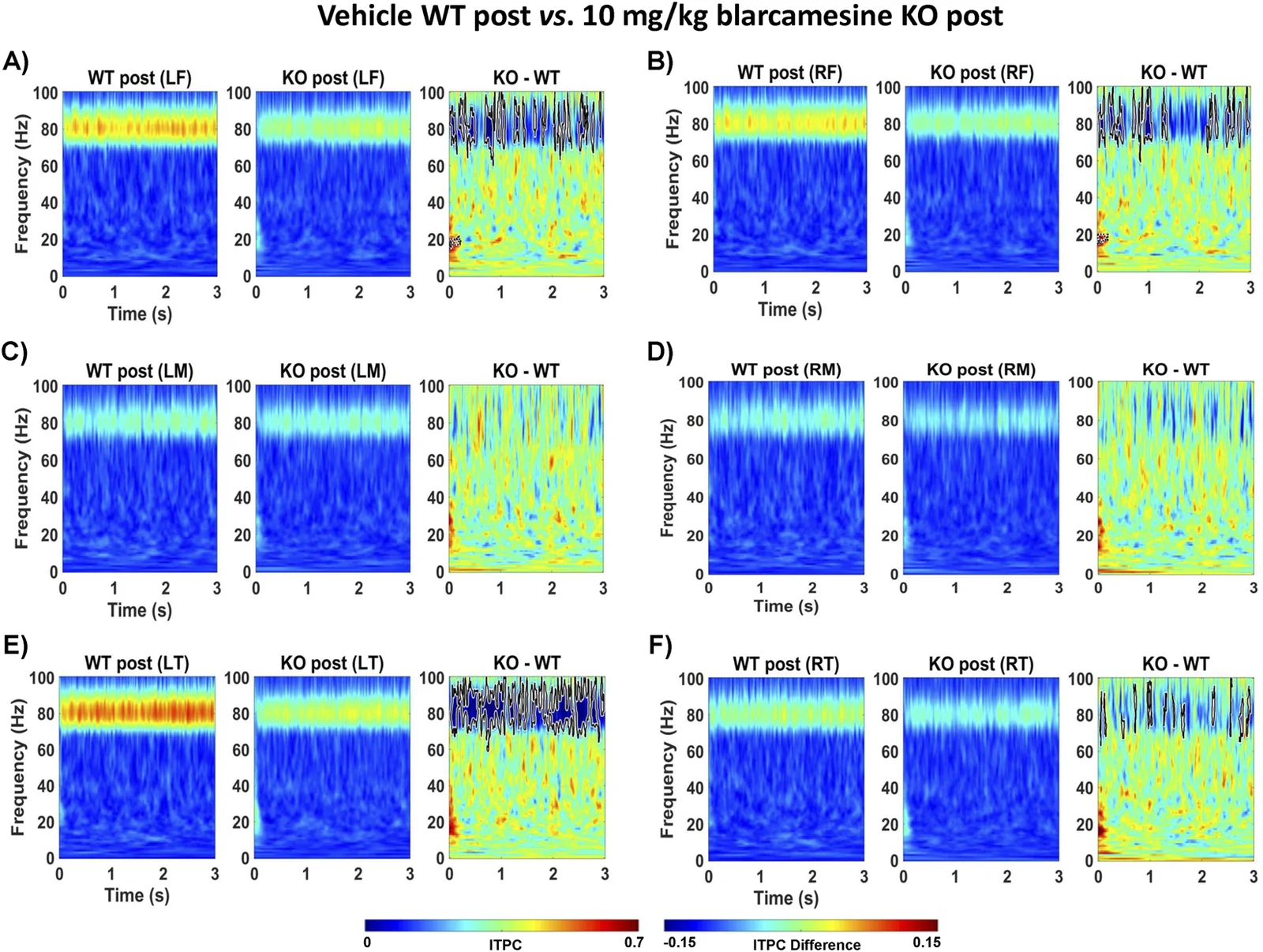
Inter-trial phase coherence (ITPC) to 80 Hz auditory steady-state response (ASSR) in vehicle-treated WT vs. 10 mg/kg blarcamesine-treated *Fmr1* KO. **(A–F)** For each brain region, the left panel shows vehicle-treated WT mice, the middle panel shows 10 mg/kg blarcamesine-treated *Fmr1* KO mice, and the right panel shows the difference (*Fmr1* KO – WT). Scales indicate ITPC and ITPC difference. Significant differences are outlined in black. Red indicates greater ITPC in 10 mg/kg blarcamesine-treated *Fmr1* KO mice relative to vehicle-treated WT mice, whereas blue indicates lower ITPC ITPC in 10 mg/kg blarcamesine-treated *Fmr1* KO mice relative to vehicle-treated WT mice. LF, left frontal; RF, right frontal; LM, left medial; RM, right medial; LT, left temporal; RT, right temporal. Vehicle-treated WT, n = 10; 10 mg/kg blarcamesine-treated Fmr1 KO, n = 15.

## Discussion

Fragile X syndrome (FXS) is the most prevalent inherited form of intellectual disability and autism spectrum disorder (Hagerman et al., 2017). FXS is also one of the best characterized neurodevelopmental disorders, which has facilitated the development of multiple therapeutic programs. Unfortunately, these have been largely unsuccessful despite encouraging preclinical data showing correction of a wide range of phenotypes in animal models (Berry-Kravis et al., 2018; Berry-Kravis et al., 2023). Although drugs targeting novel mechanisms are needed, innovative approaches for testing drugs and other therapies have also been proposed (Berry-Kravis et al., 2018). The potential of EEG parameters as biomarkers of treatment response in both affected individuals and FXS animal models has been established in studies testing different drugs (Lovelace et al., 2020; Jonak et al., 2021; Jonak et al., 2022). Here, we applied the same methodology to the evaluation of oral blarcamesine, a S1R agonist, which has already shown positive effects on several molecular and neurobehavioral paradigms in *Fmr1* KO mice (Reyes et al., 2021; Cogram et al., 2022). *Fmr1* KO mice treated with blarcamesine displayed improvements in multiple EEG parameters, known to be abnormal in this animal model, across cortical regions. These largely dose-dependent effects included decreases in resting-state gamma frequency power and increases in network synchronization (i.e., ITPC) on the chirp, 40 Hz ASSR, and 80 Hz ASSR auditory stimulation paradigms.

FXS is characterized by a variable combination of cognitive and behavioral impairments, which have a substantial impact on quality of life (Weber et al., 2019; Budimirovic et al., 2022; Lachiewicz et al., 2024). Despite the large body of knowledge on the genetics and neurobiology of FXS, and the availability of multiple animal models, no unequivocally positive effects have been found in pivotal studies of affected individuals (Berry-Kravis et al., 2018; Berry-Kravis et al., 2023). Despite targeting key pathogenetic mechanisms, several analyses of these drug development programs have suggested that the lack of preclinical measures of relevance to neurologic impairment in patients with FXS has led to unsuccessful transitions from preclinical to clinical studies (Erickson et al., 2017; Berry-Kravis et al., 2018; Grabb and Potter, 2022; Milla et al., 2023). Therefore, it is not sufficient to correct the preclinical FMRP deficiency-related phenotype but it is necessary to also demonstrate that the improvement in neurologic function could be observed in individuals with FXS (Ethridge et al., 2016; Ethridge et al., 2019). Candidate measures meeting these criteria and showing noninvasiveness and feasibility are EEG biomarkers (Ewen et al., 2019; Jonak et al., 2022). Over the years, multiple EEG parameters measured at the resting-state and after auditory stimulation have shown comparable patterns in patients with FXS and mouse models (Ethridge et al., 2016; Ethridge et al., 2017; Ethridge et al., 2019; Wang et al., 2017; Lovelace et al., 2018; Rais et al., 2018; Wen et al., 2019; Jonak et al., 2022; Jonak et al., 2024). Among these are increased resting-state gamma power and decreased synchronization of oscillations to time-varying auditory stimuli. Furthermore, these EEG parameters are associated with global (e.g., IQ, adaptive behavior, multiple aberrant behaviors) and specific (e.g., sensory hyperreactivity) cognitive and behavioral impairments, which underscores their clinical relevance (Ethridge et al., 2016; Ethridge et al., 2017; Ethridge et al., 2019). For these reasons, we conducted the present study to expand the preclinical evaluation of blarcamesine in FXS beyond traditional assessments (e.g., open field paradigm) (Reyes et al., 2021).

Consistent with our previous findings, vehicle-treated *Fmr1* KO mice exhibited significantly elevated resting-state low- and high-gamma power compared with vehicle-treated WT mice, confirming that resting-state EEG abnormalities in this model are mainly confined to the gamma frequency range (Jonak et al., 2024). In addition, vehicle-treated *Fmr1* KO mice showed reduced ITPC relative to WT mice across all three auditory stimulation paradigms, replicating the previously reported electrophysiological phenotype (Jonak et al., 2024). Following blarcamesine treatment, these electrophysiological abnormalities improved in a largely dose-dependent manner, with the greatest effects observed at 30 mg/kg across multiple cortical regions. Comparisons between blarcamesine-treated Fmr1 KO mice and vehicle-treated WT mice showed that the drug’s effects on resting-state EEG power were regionally widespread but mostly confined to the gamma frequency bands. Improvements in synchronization (i.e., increased ITPC) were observed across all cortical regions during the chirp and 40 Hz ASSR paradigms, whereas improvements during the 80 Hz ASSR paradigm were more modest and primarily restricted to the medial cortex. The greatest overall improvements were observed in 40 Hz ASSR synchronization across all blarcamesine doses, whereas the clearest dose-dependent effects were observed in the chirp paradigm. The involvement of temporal regions in synchronization improvements in the chirp and 40 Hz ASSR is not surprising since auditory stimulation would engage auditory cortical areas in the temporal region. The additional engagement of the frontal region could be interpreted as related to secondary or higher-level sensory stimulus processing, a well-established role particularly for the dorsal prefrontal cortex (Le Merre et al., 2021; Reinert et al., 2021). The changes in the medial region are more difficult to assess in terms of origin and consequences, since these recordings mainly correspond to anterior and mid cingulate areas and adjacent motor and somatosensory cortices (van Heukelum et al., 2020). Although these regions are involved in some of the same prefrontal circuits mentioned above, their physiology is less well understood.

Among the most interesting findings of relevance to FXS are those involving the low and high gamma resting-state power and the low gamma (40 Hz) ASSRs. Resting-state and evoked gamma oscillations have been linked to parvalbumin positive GABAergic interneurons function (Buzsáki and Wang, 2012; Kim et al., 2015; Hwang et al., 2019; Thankachan et al., 2019). Elevated gamma power in adult *Fmr1* KO mice, such as those studied here, may be related to alterations in inhibitory neurotransmission, consistent with multiple studies showing GABAergic dysfunction in this animal model (Hagerman et al., 2017). The ASSR paradigm is used to examine the ability of neural circuits to entrain neural oscillation to specific frequencies. It has been applied in previous mouse (Wang et al., 2020; Jonak et al., 2024) and human (Galambos et al., 1981; Kuwada et al., 1986; Aoyagi et al., 1999; Picton et al., 2003a; Picton et al., 2003b; Wong and Stapells, 2004) studies to quantify phase-locking of evoked synchronization at gamma frequencies for characterizing circuits and temporal processing (Li et al., 2024). Evoked responses to 40 Hz auditory stimuli, which peak at the same low gamma frequency across species, may reflect a cortical resonance frequency for acoustic input (Picton et al., 2003a; Picton et al., 2003b). Thus, reduced synchronization to 40 Hz ASSR stimulation may reflect abnormalities in cortical network function associated with FMRP deficiency. The 40 Hz ASSR paradigm has broad applications in the evaluation of cortical circuitry and has emerged as a biomarker in disorders such as schizophrenia (O’Donnell et al., 2013), where alterations in fast-spiking parvalbumin-positive GABAergic interneurons have also been implicated (Zhao and Guan, 2024). Reduced chirp-evoked phase locking (ITPC) in gamma frequencies, which improved following blarcamesine treatment in the present study, has also been demonstrated in individuals with FXS and correlated with increased sensory reactivity and social impairment (Ethridge et al., 2017; Ethridge et al., 2019; Wang et al., 2017). However, gamma oscillatory abnormalities are not uniquely attributable to GABAergic dysfunction and may also arise from altered excitatory drive, neuromodulatory influences, including mGluR signaling, or broader disruptions in network homeostasis. Therefore, although blarcamesine was associated with modulation of several gamma-related EEG biomarkers, the present study does not directly demonstrate rescue of parvalbumin interneuron function, GABAergic signaling, or interneuron integrity. Rather, these findings are consistent with blarcamesine influencing cortical network synchronization and circuit function through mechanisms that remain to be fully elucidated.

Because the present study did not include direct assessments of behavioral state during EEG recordings (e.g., video monitoring or locomotor activity) or a WT blarcamesine-treated group, it remains difficult to determine the extent to which these findings reflect modulation of FXS-related network abnormalities versus broader pharmacological effects associated with altered behavioral state. However, the concurrent improvements in stimulus-evoked synchronization (chirp and ASSR ITPC) together with reduced resting-state gamma power are more consistent with modulation of cortical network function than with generalized suppression of cortical activity. Nevertheless, the present study was not designed to definitively distinguish disease-specific effects from nonspecific pharmacological actions. Future studies incorporating behavioral state monitoring and drug-treated WT controls will be important for clarifying the specificity and functional significance of these electrophysiological effects.

Studies of three other drugs, namely, minocycline (Lovelace et al., 2020), the PDE10A inhibitor TAK-063 (Jonak et al., 2021) and racemic baclofen (Jonak et al., 2022), used the same general methodology presented here. When comparing the aforementioned data with the blarcamesine treatment results, it is clear that blarcamesine had more regionally widespread effects on low and high gamma power at resting state and that, unlike the other drugs, these decreases were also observed at lower doses. Similarly, blarcamesine produced dose-dependent increases in ITPC during the chirp paradigm across multiple cortical regions, with the greatest improvements observed at the intermediate and highest doses. These effects were more generalized than those previously reported for other compounds evaluated using the same paradigm (Jonak et al., 2022). In addition to improving ITPC during the chirp paradigm, blarcamesine produced dose-dependent increases in synchronization during both the 40 Hz and 80 Hz ASSR paradigms. While additional comparative analyses are needed, these initial findings suggest that blarcamesine has a favorable therapeutic index among drugs tested for EEG biomarker modulation These results should also be considered within the broader landscape of therapeutic development in FXS. Although several approaches targeting mGluR5 signaling, GABAergic neurotransmission, endocannabinoid signaling, and other downstream signaling pathways have demonstrated promising effects in preclinical studies, translation into successful clinical outcomes has remained challenging (Berry-Kravis et al., 2018; Grabb and Potter, 2022). The ability of blarcamesine to improve multiple translational EEG biomarkers associated with cortical dysfunction suggests that S1R modulation may represent a complementary therapeutic strategy for modulating dysfunction in FXS. Furthermore, because these EEG measures are directly translatable across mouse models and individuals with FXS, they may provide valuable biomarkers for evaluating pharmacodynamic responses and therapeutic efficacy in future clinical studies. Additional investigations incorporating receptor occupancy measures, downstream signaling analyses, or pharmacological validation approaches will be important to establish whether these electrophysiological changes directly reflect S1R engagement.

The study presented additional limitations to those mentioned above. The first is the use of adult animals, selected because the EEG parameters under evaluation (e.g., increased resting-state gamma power) are more evident in older than younger *Fmr1* KO mice (Jonak et al., 2024). As a neurodevelopmental disorder, drug and other treatments in FXS intend to primarily target children to improve symptoms at their more intense period and potentially modify the course of the disease. Therefore, studies similar to those conducted for baclofen, which included adolescents with FXS (Jonak et al., 2022), will be needed to assess the potential of blarcamesine for improving EEG biomarkers in younger individuals. Treatment was administered for only 2 weeks, a period that is sufficient for evaluating the reported biomarkers but perhaps insufficient for observing long-lasting behavioral effects. Nonetheless, improvements of key neurobehavioral paradigms with blarcamesine’s treatment for 2 weeks have already been reported in the *Fmr1* KO2 mouse model (Reyes et al., 2021).

In conclusion, blarcamesine improved in a dose-dependent manner in *Fmr1* KO mice multiple EEG biomarkers of relevance to FXS, including some linked to core cortical abnormalities in the disorder. These findings, in conjunction with previous studies demonstrating S1R occupancy and improvements in molecular and behavioral phenotypes following blarcamesine treatment in Fmr1 KO mice, further support the therapeutic potential of blarcamesine in FXS and other neurodevelopmental disorders. Future studies should evaluate the effects of blarcamesine on these same EEG biomarkers in individuals with FXS to determine their utility as translational pharmacodynamic biomarkers and to support the clinical development of this S1R modulator.

## Data Availability

The datasets presented in this article are not readily available. However, the datasets used and/or analyzed during the current study are available from the corresponding authors on reasonable request. Requests to access the datasets should be directed to WK (walter.e.kaufmann@emory.edu) or CJ (carrie.randle@ucr.edu).

## Supplementary material

SUPPLEMENTARY FIGURE S1 Resting-state power spectral density in vehicle-treated WT vs. vehicle-treated *Fmr1* KO mice. (A–F) Resting-state EEG power across frequency bands for distinct brain regions in vehicle-treated WT (black bars) and vehicle-treated *Fmr1* KO (white bars) mice. Values are expressed as group means, and error bars represent the standard error of the mean (SEM). Statistical significance is indicated as *p < 0.05; **p < 0.01; ***p < 0.001. Vehicle-treated WT, n = 10; vehicle-treated *Fmr1* KO, n = 10.

SUPPLEMENTARY FIGURE S2 Inter-trial phase coherence (ITPC) to auditory chirp stimulation in vehicle-treated WT vs. vehicle-treated *Fmr1* KO mice. (A–F) For each brain region, the left panel shows the averaged ITPC in vehicle-treated WT mice, the middle panel shows the averaged ITPC in vehicle-treated *Fmr1* KO mice, and the right panel shows the difference (*Fmr1* KO – WT). Scales indicate ITPC and ITPC difference. Significant differences are outlined in black. Red indicates greater ITPC in vehicle-treated *Fmr1* KO mice relative to vehicle-treated WT mice, whereas blue indicates lower ITPC in vehicle-treated *Fmr1* KO mice relative to vehicle-treated WT mice. LF, left frontal; RF, right frontal; LM, left medial; RM, right medial; LT, left temporal; RT, right temporal. Vehicle-treated WT, n = 10; vehicle-treated *Fmr1* KO, n = 10.

SUPPLEMENTARY FIGURE S3 Inter-trial phase coherence (ITPC) to 40 Hz auditory steady-state response (ASSR) vehicle-treated WT vs. vehicle-treated *Fmr1* KO mice. (A–F) For each brain region, the left panel shows the averaged ITPC in vehicle-treated WT mice, the middle panel shows the averaged ITPC in vehicle-treated *Fmr1* KO mice, and the right panel shows the difference (*Fmr1* KO – WT). Scales indicate ITPC and ITPC difference. Significant differences are outlined in black. Red indicates greater ITPC in vehicle-treated *Fmr1* KO mice relative to vehicle-treated WT mice, whereas blue indicates lower ITPC in vehicle-treated *Fmr1* KO mice relative to vehicle-treated WT mice. LF, left frontal; RF, right frontal; LM, left medial; RM, right medial; LT, left temporal; RT, right temporal. Vehicle-treated WT, n = 10; vehicle-treated Fmr1 KO, n = 10.

SUPPLEMENTARY FIGURE S4 Inter-trial phase coherence (ITPC) to 80 Hz auditory steady-state response (ASSR) vehicle-treated WT vs. vehicle-treated Fmr1 KO mice. (A–F) For each brain region, the left panel shows the averaged ITPC in vehicle-treated WT mice, the middle panel shows the averaged ITPC in vehicle-treated *Fmr1* KO mice, and the right panel shows the difference (*Fmr1* KO – WT). Scales indicate ITPC and ITPC difference. Significant differences are outlined in black. Red indicates greater ITPC in vehicle-treated *Fmr1* KO mice relative to vehicle-treated WT mice, whereas blue indicates lower ITPC in vehicle-treated *Fmr1* KO mice relative to vehicle-treated WT mice. LF, left frontal; RF, right frontal; LM, left medial; RM, right medial; LT, left temporal; RT, right temporal. Vehicle-treated WT, n = 10; vehicle-treated *Fmr1* KO, n = 10.

SUPPLEMENTARY FIGURE S5 Resting-state power spectral density in vehicle-treated WT vs. 1 mg/kg blarcamesine-treated *Fmr1* KO mice. (A–F) Ratios of WT vehicle post-treatment (black bars) to 1 mg/kg blarcamesine-treated *Fmr1* KO mice (white bars) represent EEG resting state power across frequency bands for distinct brain regions. Values are expressed as group means, and error bars represent the standard error of the mean (SEM). Statistical significance is indicated as *p < 0.05; **p < 0.01; ***p < 0.001. Vehicle-treated WT, n = 10; 1 mg/kg blarcamesine-treated *Fmr1* KO, n = 10.

SUPPLEMENTARY FIGURE S6 Inter-trial phase coherence (ITPC) to auditory chirp stimulation in vehicle-treated WT vs. 1 mg/kg blarcamesine-treated *Fmr1* KO. (A–F) For each brain region, the left panel shows vehicle-treated WT mice, the middle panel shows 1 mg/kg blarcamesine-treated *Fmr1* KO mice, and the right panel shows the difference (*Fmr1* KO – WT). Scales indicate ITPC and ITPC difference. Significant differences are outlined in black. Red indicates greater ITPC in 1 mg/kg blarcamesine-treated *Fmr1* KO mice relative to vehicle-treated WT mice, whereas blue indicates lower ITPC in 1 mg/kg blarcamesine-treated *Fmr1* KO mice relative to vehicle-treated WT mice. LF, left frontal; RF, right frontal; LM, left medial; RM, right medial; LT, left temporal; RT, right temporal. Vehicle-treated WT, n = 10; 1 mg/kg blarcamesine-treated *Fmr1* KO, n = 10.

SUPPLEMENTARY FIGURE S7 Inter-trial phase coherence (ITPC) to 40 Hz auditory steady-state response (ASSR) in vehicle-treated WT vs. 1 mg/kg blarcamesine-treated *Fmr1* KO. (A–F) For each brain region, the left panel shows vehicle-treated WT mice, the middle panel shows 1 mg/kg blarcamesine-treated *Fmr1* KO mice, and the right panel shows the difference (*Fmr1* KO – WT). Scales indicate ITPC and ITPC difference. Significant differences are outlined in black. Red indicates greater ITPC in 1 mg/kg blarcamesine-treated *Fmr1* KO, n = 10.

SUPPLEMENTARY FIGURE S8 Inter-trial phase coherence (ITPC) to 80 Hz auditory steady-state response (ASSR) in vehicle-treated WT vs. 1 mg/kg blarcamesine-treated *Fmr1* KO. (A–F) For each brain region, the left panel shows vehicle-treated WT mice, the middle panel shows 1 mg/kg blarcamesine-treated Fmr1 KO mice, and the right panel shows the difference (*Fmr1* KO – WT). Scales indicate ITPC and ITPC difference. Significant differences are outlined in black. Red indicates greater ITPC in 1 mg/kg blarcamesine-treated *Fmr1* KO mice relative to vehicle-treated WT mice, whereas blue indicates lower ITPC in 1 mg/kg blarcamesine-treated *Fmr1* KO mice relative to vehicle-treated WT mice. LF, left frontal; RF, right frontal; LM, left medial; RM, right medial; LT, left temporal; RT, right temporal. Vehicle-treated WT, n = 10; 1 mg/kg blarcamesine-treated *Fmr1* KO, n = 10.
